# Association of Serum Vaspin and Adiponectin Levels with Renal Function in Patients with or without Type 2 Diabetes Mellitus

**DOI:** 10.1155/2014/868732

**Published:** 2014-07-15

**Authors:** Meiyu Yan, Bin Su, Wenhui Peng, Liang Li, Hailing Li, Jianhui Zhuang, Yuyan Lu, Weixia Jian, Yidong Wei, Weiming Li, Shen Qu, Yawei Xu

**Affiliations:** ^1^Department of Cardiology, Endocrinology, Shanghai Tenth People's Hospital, School of Medicine, Tongji University, Shanghai 200072, China; ^2^Department of Cardiology, Shanghai Tenth People's Hospital, 301 Middle Yanchang Road, Shanghai 200072, China; ^3^Department of Endocrinology, Xinhua Hospital, School of Medicine, Shanghai Jiaotong University, Shanghai 200025, China

## Abstract

Vaspin and adiponectin are two adipocytokines with antidiabetic effects. Some studies reported that levels of adiponectin and vaspin were correlated with decreased glomerular filtration rate (FGR) and increased albuminuria. We therefore evaluated the vaspin and adiponectin levels in renal insufficiency (RI) patients with or without T2DM. Serum vaspin, adiponectin levels were measured in 416 subjects with or without T2DM. Analysis was made between groups divided by these subjects presence or absence of RI. We found that serum adiponectin level was significantly higher in nondiabetic patients with RI than in nondiabetic subjects without RI; however, there were no statistical differences between the diabetic patients with RI and without RI. In all the subjects, the serum adiponectin level was also higher in 50 individuals with RI than that in 366 subjects without RI. The serum vaspin levels showed no significant differences between the diabetic patients or nondiabetics subjects with RI and without RI. Contrary to adiponectin, the serum vaspin level was lower in 169 patients with T2DM than in 247 individuals without T2DM. Our data suggested that both of T2DM and renal insufficiency were correlated with the serum level of adiponectin. However, the serum vaspin levels showed no significant difference between the individuals with renal insufficiency and without renal insufficiency.

## 1. Introduction

Visceral adipose tissue-derived serine proteinase inhibitor (vaspin) was identified in the visceral adipose tissue of OLETF (Otsuka Long-Evans Tokushim a Fatty) rats, an animal model of obesity and type 2 diabetes mellitus (T2DM) [1]. Vaspin is also expressed in the skin, hypothalamus, pancreatic islets, and stomach [2]. Circulating levels of adiponectin, a hormone produced predominantly by adipocytes, are highly heritable and are inversely associated with T2DM and other metabolic traits [3]. Both of the two adipocytokines are associated with the diabetes and other metabolic disorders. Some studies have shown that plasma vaspin concentrations are significantly higher in men with the metabolic syndrome compared with those without the metabolic syndrome [4]. The serum vaspin levels in the patients with type 2 diabetes have been demonstrated to be higher than [5, 6] or similar to [7] those observed in subjects with normal glucose tolerance. In recent, Inoue et al. reported that the serum vaspin levels are negatively correlated with the creatinine levels and were significantly reduced in the Japanese chronic hemodialysis (HD) patients [8]. Thus, it was interesting to ask whether serum vaspin levels are elevated or reduced in diabetic patients with renal insufficiency. We measured serum vaspin and adiponectin levels in subjects with normal renal function and renal insufficiency with or without T2DM and compared various clinical parameters.

## 2. Materials and Methods

### 2.1. Subjects

All subjects were local residents of Han ethnicity in Shanghai and consecutively recruited in Department of Endocrinology and Cardiology, Xinhua Hospital and Shanghai Tenth People's Hospital from June 2011 to December 2011. The study was approved by the Ethical Committee of Shanghai Tenth People's Hospital and Xinhua Hospital. Clinical characteristics were recorded with respect to age, sex, body mass index (BMI), risk factors for renal function, and diabetic mellitus (DM). DM was defined as fasting serum glucose level ≥7.0 mmol/L or 2-hour postprandial glucose ≥11.1 mmol/L, or taking hypoglycemic drugs. Those with type 1 diabetes, acute myocardial infarction, systemic inflammatory disorders, severe heart failure (left ventricular ejection fraction <30%), advanced renal insufficiency, and malignant tumor were excluded.

### 2.2. ELISA Assays and Biochemical Investigations

Blood samples were centrifuged at 1000 g for 10 minutes. Plasma specimens were then frozen and stored at −80°C until analysis. Human total Adiponectin (R&D Systems, Minneapolis, American) and Vaspin (Adipogen, Seoul, South Korea) plasma levels were measured with commercially available ELISAs as indicated before [9]. While high-sensitive C-reactive protein (hsCRP), fibrinogen (FIB), creatinine (Cr), blood urea nitrogen (BUN), uric acid (UA), fasting blood glucose (FBG), 2-hour postprandial glucose (PG2h), hemoglobin A1c (HbA1c), and lipid profiles, including total cholesterol (TC), triglyceride (TG), high-density lipoprotein cholesterol (HDL-C), low-density lipoprotein cholesterol (LDL-C), apolipoprotein A (ApoA), and apolipoprotein B (ApoB) were measured by colorimetric enzymatic assay systems (Roche MODULAR P-800, Swiss Confederation).

### 2.3. Patients

416 subjects were divided into 4 groups according to the presence or absence of RI and T2DM: 247 individuals without T2DM who had RI (*n* = 27; Group I) or normal renal function (*n* = 220; Group II) and 169 patients with T2DM who had RI (*n* = 23; Group III) or normal renal function (*n* = 146; Group IV).

### 2.4. Renal Function Evaluation

Renal function was assessed by the estimated creatinine clearance (CrCl) derived from Cockroft-Gault formula, where CrCl (mL/min) = ([140 − age (years)] × weight (kg))/(0.818 × serum creatinine (*μ*mol/L)), corrected in women by a factor of 0.85 [10]. A calculated CrCl less than 60 mL/min was defined as RI, according to guidelines [11].

### 2.5. Statistical Analysis

Statistical analyses were performed using the PASW Statistics 20 software (IBM Corporation, Armonk, NY, USA). Normality of distribution was evaluated using the Kolmogorov-Smirnov test. Comparisons of variables with a normal distribution were made using Student's *t*-test, and values were provided as mean ± S.D. For parameters with an abnormal distribution, the Mann-Whitney *U* test was used for comparisons, and values were given as median (interquartile range). Categorical variables were presented as frequencies. To evaluate the correlation between serum vaspin and adiponectin levels with other variables such as age, BMI, hsCRP, Crcl, Cr, FBG, and TC single linear univariate correlations and multivariate regression analyses were performed. A 2-sided probability level of ≤0.05 was taken as significance.

## 3. Results and Discussion

### 3.1. Results

#### 3.1.1. Clinical Characteristics

In the population either with T2DM or without T2DM, patients with RI (Group I or Group III) were older and had lower BMI, higher systolic blood pressure (SBP) than those without RI (Group II or Group IV) (all *P* < 0.05) (Table 1).

#### 3.1.2. The Serum Vaspin and Adiponectin Levels in the Four Groups

The serum vaspin showed no significant differences either between non-T2DM patients with RI (Group I) and without RI (Group II) (0.548 (0.428–1.136) ng/mL versus 0.517 (0.232–1.324) ng/mL, *P* = 0.402) or between diabetic patients with RI (Group III) and without (Group IV) (0.497 (0.288–0.907) ng/mL versus 0.381 (0.232–0.740) ng/mL, *P* = 0.245). Interestingly, the serum vaspin was lower in 169 patients with T2DM than in 247 individuals without T2DM (0.399 (0.224–0.737 ng/mL versus 0.523 (0.182–1.172) ng/mL, *P* < 0.05) (Table 2). However, no significant difference for the serum vaspin level was observed between 50 patients with RI (combined by Group I and Group III) and 366 individuals without RI (combined by Group II and Group IV) (0.540 (0.399–1.020) ng/mL versus 0.432 (0.234–0.925) ng/mL, *P* = 0.141) (Table 2).

Compared with the nondiabetic individuals without RI (Group II), the serum adiponectin was higher in the non-diabetic individuals with RI (Group I) (16082.437 ± 9926.203 ng/mL versus 7824.135 ± 7032.657 ng/mL, *P* < 0.01) but did not differ between diabetic patients with RI (Group III) and without RI (Group IV) (12987.336 ± 14623.762 ng/mL versus 8744.920 ± 5628.952 ng/mL, *P* = 0.349). Regardless of the individuals with or without T2DM, the serum adiponectin level was significantly higher in 50 individuals with RI (combined by Group I and III) than 366 individuals without RI (combined by Group II and IV) (10549.706 (5589.718–22429.025) ng/mL versus 6955.130 (2590.052–13533.955) ng/mL, *P* < 0.01) (Tables 1 and 2). When compared with the individuals without T2DM (combined by Group I and II) in our tall 416 samples, the serum adiponectin was significantly higher in 169 patients with T2DM (combined by Group III and IV) (7599.068 (4100.633–13827.261) ng/mL versus 5553.476 (12.096–14225.162) ng/mL, *P* < 0.05).

#### 3.1.3. Univariate Correlations of Vaspin and Adiponectin with Other Parameters

Spearmen's analysis showed that serum vaspin level was only related to Cr in patients without T2DM. In patients with T2DM, serum vaspin levels were related to age, SBP, and UA. In all subjects, serum vaspin levels were related to Crcl, TC, and HbA1c (Tables 3 and 4).

The serum adiponectin levels were related to age, SBP, hsCRP, Crcl, and Cr in subjects without T2DM. In patients with T2DM, serum adiponectin levels were related to FBG, LDL-C. In all subjects, serum adiponectin levels were related to age, SBP, Crcl, Cr, BUN, and HbA1c (Tables 3 and 4).

#### 3.1.4. Multivariate Correlations of Vaspin and Adiponectin with Other Parameters

Multiple regression analyses were performed to test whether any of the following factors influence the serum vaspin and adiponectin concentrations: age, SBP, FBG, UA, Crcl, Cr, TG, TC, HbA1c levels, and so on. When correcting for these variables using stepwise regression analyses after adjustment for age and BMI, the level of HbA1c was found to be independently and significantly associated with the vaspin concentration in 427 total subjects. However, no significant correlations between the serum vaspin level and any parameter were detected in both groups of the subjects with T2DM and without T2DM. The level of Crcl was found to be independently and significantly associated with the adiponectin concentration in all subjects. However, in the groups of the subjects with or without T2DM, no significant correlations were detected between the serum adiponectin level and any parameter (Tables 5 and 6).

#### 3.1.5. Comparisons of Serum Vaspin and Adiponectin Divided by Gender

Mann-Whitney *U* test showed that the serum vaspin and adiponectin levels were both higher in women than in men whether in total subjects or in patients with T2DM (Tables 7 and 8).

### 3.2. Discussion

Adipose tissue is a highly active endocrine organ secreting a number of bioactive molecules called adipokines [5, 6, 12], such as vaspin and adiponectin. Vaspin is a member serpin A12 of the serine protease inhibitor family [13–15], and a large number of clinical data have showed that vaspin played an important role in occurrence and development in the metabolic syndrome [1, 16, 17]. Upregulation of vaspin can improve the insulin resistance [17], which also inhibit inflammation and progression of arthrosclerosis [18]. The mechanism of vaspin and the downstream signal pathway are not fully clear. Some studies show that vaspin increases nitric oxide bioavailability through the reduction of asymmetric dimethylarginine in vascular endothelial cells [19] or inhibits platelet-derived growth factor-BB-induced migration of vascular smooth muscle cells [20]. The decrease of vaspin in the RI patients will increase the risk of coronary heart disease (CHD). Therefore, measuring the levels of adipokines in patients with RI may be beneficial for predicting cardiovascular events and patient survival.

In patients with CKD, the levels of adipokines appear to increase in association with declines in the GFR. This is most likely due to reduced renal metabolism of adipokines, which may not increase vascular risks in patients with a reduced renal function [21]. Since vaspin is a small protein size with the molecular mass 50 kDa, which would be freely filtered by the kidneys [8]; thus it is reasonable to presume that vaspin levels should be increased in patients with renal insufficiency. But our data suggested that the serum vaspin levels have no significant difference between patients with RI and subjects without RI due to the fact that many factors might affect the serum levels of vaspin concentration.

Previous studies have shown that, in the diabetic patients, serum vaspin level is higher than that in normal glucose tolerance subjects in women [22], and rosiglitazone therapy decreased plasma vaspin levels through glucose and insulin sensitivity regulation [23]. Our study also shows that the serum vaspin level was higher in women than in men. However, we have found that the serum vaspin level was lower in 169 diabetic patients than 247 individuals without T2DM. The paradox results may be caused by two reasons: one is the sex difference in the subjects between their study and our study; another reason is that there are differences in the diabetic patients with vascular complications between the two population, since some reports have demonstrated that low vaspin serum concentrations have been found to be correlated with recently experienced ischemic events in patients with carotid stenosis [13] and coronary artery disease (CAD) [24].

Adiponectin is a fat-derived hormone whose reduction plays central roles in obesity-linked diseases including insulin resistance, type 2 diabetes, and atherosclerosis. Adiponectin multimers are secreted by white adipose tissue [25–27], which is associated with body weight and markers of cardiovascular risk in adolescence [28]. Adiponectin has recently attracted much attention because of its antidiabetic and antiatherogenic effects as well as its antiproliferative effects in cancer cells; thus, adiponectin is expected to be a therapeutic tool for diabetes, metabolic syndrome, cardiovascular diseases, and cancers [29, 30]. Plasma adiponectin levels have also been reported to be reduced in obese humans, particularly those with visceral obesity [31, 32]. So, in the RI patients, lower BMI may lead to the high level of adiponectin. Another reason may be the poor filtration function of the renal. But in the diabetic patients, there are no differences between RI and non-RI. Some studies have shown that rosiglitazone can increase serum adiponectin levels in patients with type 2 diabetes mellitus [33, 34]. In all patients with diabetes in our current study, only 19 patients have been treated with thiazolidinediones. After removing the patients, the results have no significant change.

## 4. Conclusions

Both of T2DM and renal insufficiency were correlated with the serum level of adiponectin. Although the serum vaspin levels showed no significant difference between the patients with renal insufficiency which did not required treatment by chronic hemodialysis and subjects without renal insufficiency, we have found that the serum vaspin level was lower in 169 diabetic patients than in 247 individuals without T2DM.

## Figures and Tables

**Table 1:** Baseline of all subjects divided by T2DM and RI.

	Non-T2DM			T2DM		
	RI	Non-RI	*P* value	RI	Non-RI	*P* value
Gender (male : female)	9 : 18	151 : 69		13 : 10	75 : 71	
Age (years)	73.85 ± 9.02	53.19 ± 17.13	<0.001∗	75.70 ± 7.38	61.03 ± 10.50	<0.001∗
BMI (kg/m^2^)	22.01 ± 2.51	24.17 ± 3.71	<0.01∗	22.90 ± 3.17	25.56 ± 4.49	<0.01∗
SBP (mmHg)	149.70 ± 26.89	135.05 ± 24.13	<0.01∗	147.78 ± 21.92	136.92 ± 19.80	<0.05∗
DBP (mmHg)	82.00 ± 10.47	78.64 ± 13.33	0.227	79.96 ± 11.98	79.59 ± 11.17	0.886
Vaspin (ng/mL)	0.55 (0.43–1.14)	0.52 (0.23–1.32)	0.402	0.50 (0.29–0.91)	0.38 (0.23–0.74)	0.245
Adiponectin (ng/mL)	16082.44 ± 9926.20	7824.135 ± 7032.657	<0.01∗	12987.34 ± 14623.76	8744.92 ± 5628.95	0.349
hsCRP (mg/L)	3.27 (3.10–3.27)	3.34 (2.93–7.05)	0.266	1.85 (0.05–6.21)	1.01 (0.06–3.06)	0.428
Crcl (mL/min)	48.01 ± 10.43	95.94 ± 36.92	<0.001∗	46.24 ± 10.45	112.58 ± 43.43	<0.001∗
Cr (*μ*mol/L)	92.48 ± 28.11	73.84 ± 16.29	<0.01∗	100.04 ± 26.65	59.68 ± 16.78	<0.001∗
BUN (mmol/L)	6.26 ± 2.36	5.37 ± 1.36	0.068	8.46 ± 3.75	5.61 ± 1.69	<0.01∗
UA (*μ*mol/L)	392.41 ± 80.61	339.41 ± 85.37	<0.01∗	388.96 ± 96.66	288.86 ± 81.32	<0.001∗
FBG (mmol/L)	4.96 ± 0.63	5.05 ± 0.74	0.576	7.40 ± 2.41	7.91 ± 2.95	0.498
PG2h (mmol/L)	7.11 ± 2.02	7.14 ± 2.32	0.974	12.06 ± 3.58	12.53 ± 4.41	0.732
HbA1c (%)	5.13 ± 1.75	5.66 ± 0.40	0.342	8.86 ± 1.81	8.66 ± 2.10	0.709
TC (mmol/L)	4.89 ± 1.05	4.48 ± 1.25	0.124	4.40 ± 1.07	4.69 ± 1.07	0.242
TG (mmol/L)	1.41 ± 0.64	1.45 ± 0.83	0.797	1.32 (0.99–1.63)	1.51 (1.09–2.53)	0.131
HDL-C (mmol/L)	1.30 ± 0.36	1.18 ± 0.30	0.107	1.13 ± 0.42	1.20 ± 0.33	0.387
LDL-C (mmol/L)	2.80 ± 0.85	2.59 ± 0.99	0.326	2.62 ± 1.05	2.71 ± 0.72	0.627
ApoA (g/L)	1.32 ± 0.16	1.26 ± 0.15	0.314	1.29 ± 0.26	1.31 ± 0.25	0.738
ApoB (g/L)	0.90 ± 0.17	1.12 ± 0.81	0.350	0.87 (0.70–1.02)	0.96 (0.77–1.22)	0.170
LPA (mg/L)	169.25 ± 74.190	174.46 ± 106.72	0.877	5.55 (2.80–53.00)	5.90 (1.14–19.83)	0.229
FIB (g/L)	2.56 ± 0.51	2.37 ± 0.65	0.206	2.78 ± 0.64	3.09 ± 0.83	0.132

The data are presented as the number, mean ± S.D., or median (interquartile range); *P < 0.05.

BMI: body mass index; SBP: systolic blood pressure; DBP: diastolic blood pressure; vaspin: visceral adipose-tissue-derived serpin; hsCRP: high-sensitive C-reactive protein; Crcl: creatinine clearance; Cr: creatinine; BUN: blood urea nitrogen; UA: uric acid; FBG: fasting blood glucose; PG2h: 2-hour postprandial glucose; HbA1c: hemoglobin A1c; TG: triglyceride; TC: total cholesterol; HDL-C: high-density lipoprotein cholesterol; LDL-C: low-density lipoprotein cholesterol; ApoA: apolipoprotein A; ApoB: apolipoprotein B; LPA: lipoprotein A; FIB: fibrinogen.

**Table 2:** Comparisons of serum vaspin and adiponectin divided by T2DM and RI.

	Non-T2DM	T2DM	*P* value
Vaspin	0.52 (0.18–1.17)	0.40 (0.22–0.74)	<0.05
Adiponectin	5553.48 (12.10–14225.16)	7599.07 (4100.63–13827.26)	<0.05

	Non-RI	RI	*P* value

Vaspin	0.43 (0.23–0.93)	0.54 (0.40–1.02)	0.141
Adiponectin	6955.13 (2590.05–13533.96)	10549.71 (5589.72–22429.03)	<0.01

**Table 3:** Single univariate linear correlations of factors associated with serum vaspin levels.

	Non-T2DM		T2DM		All subjects	
	*r*	*P* value	*r*	*P* value	*r*	*P* value
Age	0.033	0.626	0.175	0.030∗	0.043	0.408
BMI	0.068	0.436	0.112	0.205	0.070	0.258
SBP	0.009	0.899	0.195	0.016∗	0.068	0.189
DBP	−0.025	0.717	0.129	0.112	0.034	0.516
Adiponectin	0.118	0.098	0.163	0.302	0.126	0.052
hsCRP	−0.078	0.677	−0.097	0.323	−0.139	0.106
Crcl	−0.094	0.311	−0.166	0.054	−0.138	0.027∗
Cr	−0.181	0.009∗	0.051	0.535	−0.044	0.412
BUN	0.017	0.810	0.067	0.411	0.029	0.574
UA	−0.075	0.297	0.160	0.050∗	0.053	0.329
PG2h	−0.011	0.930	−0.118	0.365	−0.124	0.169
HbA1c	−0.100	0.415	−0.140	0.107	−0.209	0.003∗
TC	0.105	0.127	0.100	0.233	0.105	0.047∗
TG	0.095	0.164	0.026	0.753	0.042	0.433
HDL-C	−0.015	0.831	0.051	0.541	0.007	0.900
LDL-C	0.125	0.084	0.007	0.931	0.075	0.170
ApoA	0.122	0.344	−0.009	0.921	0.022	0.770
ApoB	0.152	0.238	0.010	0.915	0.051	0.496
LPA	−0.196	0.147	0.020	0.832	0.069	0.367
FIB	0.086	0.340	−0.182	0.051	−0.066	0.306

*P < 0.05. BMI: body mass index; SBP: systolic blood pressure; DBP: diastolic blood pressure; vaspin: visceral adipose-tissue derived serpin; hsCRP: high-sensitive C-reactive protein; Crcl: creatinine clearance; Cr: creatinine; BUN: blood urea nitrogen; UA: uric acid; PG2h: 2-hour postprandial glucose; HbA1c: hemoglobin A1c; TG: triglyceride; TC: total cholesterol; HDL-C: high-density lipoprotein cholesterol; LDL-C: low-density lipoprotein cholesterol; ApoA: apolipoprotein A; ApoB: apolipoprotein B; LPA: lipoprotein A; FIB: fibrinogen.

**Table 4:** Single univariate linear correlations of factors associated with serum adiponectin levels.

	Non-T2DM		T2DM		All subjects	
	*r*	*P* value	*r*	*P* value	*r*	*P* value
Age	0.614	<0.001∗	0.183	0.245	0.589	<0.001∗
BMI	0.081	0.357	0.164	0.317	0.093	0.224
SBP	0.170	0.018∗	0.104	0.511	0.177	0.007∗
DBP	−0.057	0.432	−0.074	0.641	−0.052	0.432
Vaspin	0.118	0.098	0.163	0.302	0.126	0.052
hsCRP	−0.543	0.030∗	0.213	0.555	−0.172	0.402
Crcl	−0.526	<0.001∗	−0.098	0.552	−0.434	<0.001∗
Cr	0.198	0.007∗	0.018	0.911	0.160	0.017∗
BUN	0.140	0.054	0.202	0.206	0.157	0.017∗
UA	0.054	0.482	0.050	0.758	0.030	0.661
PG2h	−0.157	0.271	−0.151	0.443	−0.166	0.143
HbA1c	−0.078	0.575	−0.245	0.218	−0.254	0.022∗
TC	−0.106	0.147	0.318	0.055	−0.049	0.467
TG	0.023	0.754	−0.315	0.058	0.023	0.726
HDL-C	0.116	0.134	0.157	0.360	0.093	0.186
LDL-C	−0.014	0.852	0.339	0.040∗	0.036	0.613
ApoA	0.186	0.173	−0.138	0.519	0.102	0.372
ApoB	−0.016	0.906	0.223	0.305	0.033	0.773
LPA	0.133	0.357	0.235	0.319	0.179	0.138
FIB	−0.010	0.915	0.272	0.094	0.038	0.646

*P < 0.05. BMI: body mass index; SBP: systolic blood pressure; DBP: diastolic blood pressure; vaspin: visceral adipose-tissue-derived serpin; hsCRP: high-sensitive C-reactive protein; Crcl: creatinine clearance; Cr: creatinine; BUN: blood urea nitrogen; UA: uric acid; PG2h: 2-hour postprandial glucose; HbA1c: hemoglobin A1c; TG: triglyceride; TC: total cholesterol; HDL-C: high-density lipoprotein cholesterol; LDL-C: low-density lipoprotein cholesterol; ApoA: apolipoprotein A; ApoB: apolipoprotein B; LPA: lipoprotein A; FIB: fibrinogen.

**Table 5:** Multivariate linear regression analyses of all subjects, patients with and without T2DM, using the serum vaspin levels as dependent variables.

Models	Independent variables	*B*	Beta	*T* value	*P* value	Model *r* ^2^
1	All subjects					
	Age	−0.023	−0.024	−0.286	0.775	0.044
	BMI	−0.046	−0.047	−0.573	0.568	
	Crcl	−0.061	−0.061	−0.738	0.461	
	TC	0.071	0.072	0.883	0.379	
	HbA1c	−0.235	−0.209	−2.612	0.010∗	

2	Non-T2DM					
	Age	0.026	0.121	1.194	0.235	0.017
	BMI	−0.024	−0.022	−0.225	0.823	
	Cr	−0.019	−0.102	−1.027	0.307	

3	T2DM					
	Age	0.008	0.110	0.949	0.346	0.066
	BMI	0.025	0.141	1.224	0.224	
	FBG	−0.042	−0.149	−1.308	0.194	
	UA	0.001	0.133	1.172	0.244	

*P < 0.05. BMI: body mass index; Crcl: creatinine clearance; Cr: creatinine; UA: uric acid; FBG: fasting blood glucose; HbA1c: hemoglobin A1c; TC: total cholesterol.

**Table 6:** Multivariate linear regression analyses of all subjects, patients with and without T2DM, using the serum adiponectin levels as dependent variables.

Models	Independent variables	*B*	Beta	*T* value	*P* value	Model *r* ^2^
1	All subjects					
	Age	0.196	0.154	1.226	0.225	0.132
	BMI	−0.154	−0.141	−1.120	0.267	
	SBP	−0.070	−0.074	−0.582	0.563	
	Crcl	−86.311	−0.363	−3.089	0.003∗	
	Cr	−0.248	−0.220	−1.779	0.080	
	BUN	−0.106	−0.107	−0.848	0.400	
	HbA1c	−0.175	−0.184	−1.475	0.145	

2	Non-T2DM					
	Age	79.928	0.203	0.215	0.837	0.384
	BMI	−810.955	−0.320	−0.471	0.654	
	SBP	−190.316	−0.475	−1.088	0.318	
	Crcl	−93.939	−0.353	−0.353	0.736	
	Cr	−89.348	−0.248	−0.364	0.728	
	hsCRP	12.961	0.085	0.211	0.840	

3	T2DM					
	Age	231.786	0.215	1.204	0.239	0.121
	BMI	151.377	0.066	0.323	0.749	
	FBG	−611.036	−0.190	−0.925	0.363	
	LDL-C	1778.647	0.186	1.012	0.320	

*P < 0.05. BMI: body mass index; SBP: systolic blood pressure; hsCRP: high-sensitive C-reactive protein; Crcl: creatinine clearance; Cr: creatinine; BUN: blood urea nitrogen; FBG: fasting blood glucose; HbA1c: hemoglobin A1c; LDL-C: low-density lipoprotein cholesterol.

**Table 7:** Comparisons of serum vaspin and adiponectin levels divided by gender.

Models	Men	Women	*P* value
Vaspin	1.07	1.72	<0.01
Adiponectin	7063.03	11866.85	<0.01

**Table 8:** Comparisons of serum vaspin and adiponectin levels divided by gender and T2DM.

Models	T2DM			Non-T2DM		
	Men	Women	*P* value	Men	Women	*P* value
Vaspin	0.51	0.74	<0.01	1.38	2.73	<0.01
Adiponectin	7367.09	12419.30	0.021	7015.52	11695.67	<0.01
